# Synthesis and characterization of electrospun molybdenum dioxide–carbon nanofibers as sulfur matrix additives for rechargeable lithium–sulfur battery applications

**DOI:** 10.3762/bjnano.9.28

**Published:** 2018-01-24

**Authors:** Ruiyuan Zhuang, Shanshan Yao, Maoxiang Jing, Xiangqian Shen, Jun Xiang, Tianbao Li, Kesong Xiao, Shibiao Qin

**Affiliations:** 1 Institute for Advanced Materials, College of Materials Science and Engineering, Jiangsu University, Zhenjiang, 212013, P. R. China; 2 Hunan Engineering Laboratory of Power Battery Cathode Materials, Changsha Research Institute of Mining and Metallurgy, Changsha, 412212, P. R. China; 3 School of Mathematics and Physics, Jiangsu University of Science and Technology, Zhenjiang, 212013, P. R. China

**Keywords:** electrochemical performance, electrospinning, lithium–sulfur batteries, MoO_2_–CNFs, sulfur matrix

## Abstract

One-dimensional molybdenum dioxide–carbon nanofibers (MoO_2_–CNFs) were prepared using an electrospinning technique followed by calcination, using sol–gel precursors and polyacrylonitrile (PAN) as a processing aid. The resulting samples were characterized by X-ray diffraction (XRD), Fourier transform infrared spectroscopy (FTIR), Raman spectroscopy, Brunauer–Emmet–Teller (BET) surface area measurements, scanning electron microscopy (SEM) and transmission electron microscopy (TEM). MoO_2_–CNFs with an average diameter of 425–575 nm obtained after heat treatment were used as a matrix to prepare sulfur/MoO_2_–CNF cathodes for lithium–sulfur (Li–S) batteries. The polysulfide adsorption and electrochemical performance tests demonstrated that MoO_2_–CNFs did not only act as polysulfide reservoirs to alleviate the shuttle effect, but also improve the electrochemical reaction kinetics during the charge–discharge processes. The effect of MoO_2_–CNF heat treatment on the cycle performance of sulfur/MoO_2_–CNFs electrodes was examined, and the data showed that MoO_2_–CNFs calcined at 850 °C delivered optimal performance with an initial capacity of 1095 mAh g^−1^ and 860 mAh g^−1^ after 50 cycles. The results demonstrated that sulfur/MoO_2_–CNF composites display a remarkably high lithium–ion diffusion coefficient, low interfacial resistance and much better electrochemical performance than pristine sulfur cathodes.

## Introduction

Lithium–sulfur (Li–S) batteries are considered to be the most promising candidates for the next green rechargeable batteries due to their high energy density (2600 Wh kg^−1^) and theoretical specific capacity (1675 mAh g^−1^). However, before Li–S batteries become a viable technology, some challenges need to be solved such as the insulating nature of sulfur and the shuttle effect caused by dissolved polysulfide molecules [1]. All of these issues still pose a challenge to overcome for the production of reversible, stable, and efficient sulfur cathodes. The currently proposed approaches to solve these issues include sulfur-based cathode modification, electrolyte modification and new cell configuration [2].

Overall, it is critical to enhance the utilization of sulfur and stabilize the polysulfide within the cathodic region to yield Li–S batteries with improved electrochemical performance. For the past two decades, various carbon materials (e.g., mesoporous carbon [3], multiwalled carbon nanotubes (MWNTs) [4] and hollow carbon microspheres [5]) and electrically conductive polymeric materials (e.g., polyaniline [6], polypyrrole [7] and poly(3,4-ethylenedioxythiophene) [8]) have been considerably used to encapsulate sulfur or polysulfide. Recently, polar metal oxide/sulfide materials including SiO_2_ [9], TiO_2_ [10], MnO_2_ [11], Mg_0.6_Ni_0.4_O [12], TiS_2_ [13], CoS_2_ [14], and FeS_2_ [15] were found to be more highly effective in binding with sulfur species than carbon substrates, and were found to significantly improve the cycling behavior of Li–S batteries. However, these metal oxide/sulfide materials have low electrical conductivity, which makes the chemically adsorbing polysulfides difficult to be reduced directly on the matrix surface, resulting in the lower reutilization of the active sulfur material. In order to improve the kinetics of the electrode redox reaction, the electrical conductivity of Ti_4_O_7_ nanoparticles [16–17] and Co_9_S_8_ [18] nanosheets have been used in new concepts for sulfur matrices with both a polar nature and good conductivity. Therefore, the exploration of novel conductive composites is another direction leading to the practical application of Li–S batteries.

Molybdenum dioxide (MoO_2_) materials are particularly attractive among the transition-metal oxides due to their high melting point, high chemical stability and low electrical resistivity (190 S cm^−1^). This material has great potential for applications in several fields such as sensing, catalysis, supercapacitors and as an anode material in lithium ion batteries due to its relatively large theoretical capacity [19–21]. Although numerous synthetic approaches have been reported for preparing MoO_2_ nanostructures with diverse morphologies, the fabrication, manipulation, and engineering of one-dimensional (1D) MoO_2_–CNFs nanocomposites, especially with secondary MoO_2_ nanostructures, are difficult to achieve due to lack of appropriate and generalized synthetic methodologies. Recently, hierarchical MoO_2_/C microspheres and hierarchical MoO_2_/Mo_2_C/C hybrid nanowires were fabricated using organic–inorganic precursors and self-templates, which were used as anode materials in lithium ion batteries [22–23]. However, since electrospinning is a simple and versatile method for producing fibers from a variety of materials on a large scale, it has attracted much attention in both research and commerce [24]. The nanofibers have extremely high specific surface area because of their small diameter and their porosity which exhibits excellent pore interconnectivity [25–26]. To the best of our knowledge, no articles related to using MoO_2_–CNFs as a sulfur matrix in Li–S batteries have been published so far.

In the present work, a facile route based on a single-spinneret electrospinning technique with a subsequent annealing process was developed to prepare MoO_2_–CNFs. The effect of MoO_2_–CNF heat treatment on the cycle performance of sulfur/MoO_2_–CNFs electrodes was examined. The data showed that MoO_2_–CNFs calcined at 850 °C delivered optimal performance, with an initial capacity of 1095 mAh g^−1^ and retained a capacity of 860 mAh g^−1^ after 50 cycles. The results demonstrated that the sulfur/MoO_2_–CNF composite displays a markedly high lithium-ion diffusion coefficient, a low interfacial resistance and much better electrochemical performance than a pristine sulfur cathode.

## Results and Discussion

### Characterization of MoO_2_–CNFs

**Figure 1:**
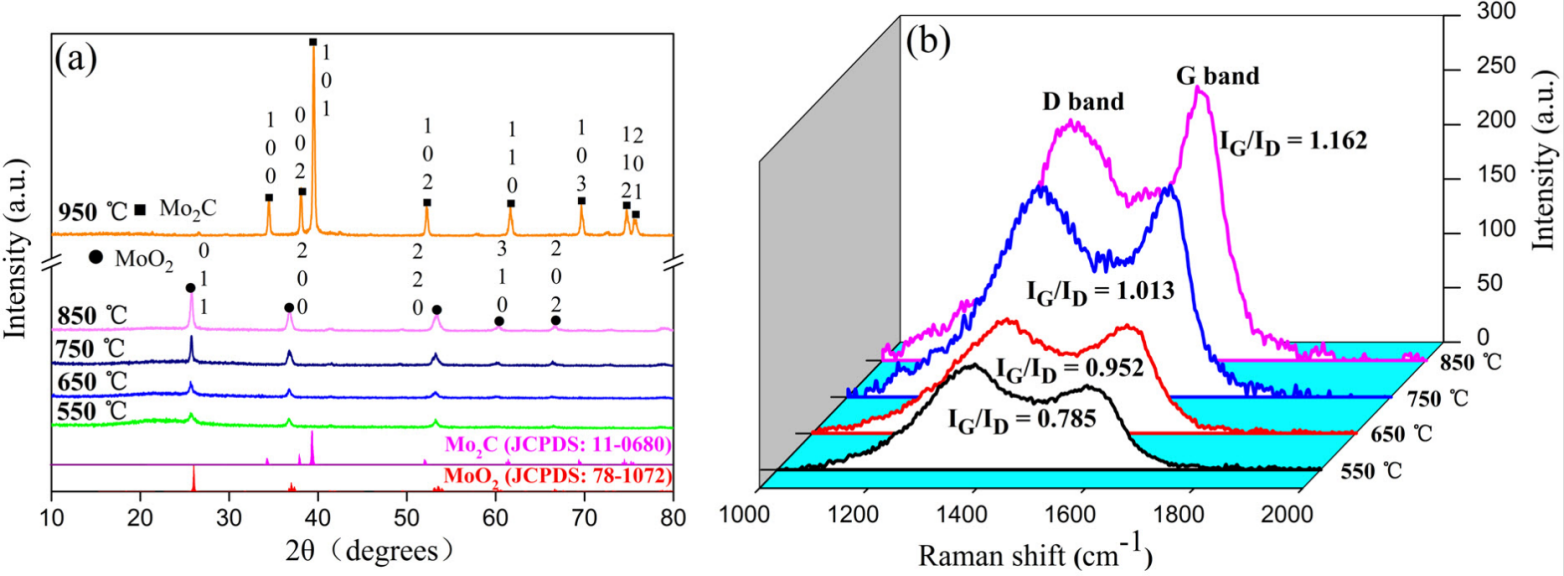
(a) XRD pattern and (b) Raman spectra of the MoO_2_–CNFs calcined at various temperatures.

X-ray diffraction (XRD) patterns of the as-prepared composite fibers calcined at various temperatures are presented in Figure 1a. Well-defined features appeared for the samples heated at 550 °C due to the crystallization of MoO_2_. Five main peaks at 2θ of 25.8°, 36.8°, 53.4°, 60.4° and 66.7° were assigned to the crystallographic planes of (011), (200), (220), (310) and (202), respectively. These corresponded to pure phase MoO_2_ with monoclinic symmetry, which agreed well with the JCPDS card of MoO_2_ (78-1072). As the calcination temperature was raised to 850 °C, the characteristic diffraction peaks of MoO_2_ became sharper and displayed higher intensities, indicating an increase in the crystallinity of MoO_2_ as shown in Table 1. Meanwhile, this was also reflected in the surface area, which decreased as the calcination temperature increased (Table 1).

**Table 1:** Effect of calcination temperature on BET surface area and particle size of MoO_2_.

Calcination temperature (°C)	BET surface area (m^2^ g**^−1^**)	Particle size (nm)^a^

550	312.65	42.93
650	226.30	50.67
750	182.33	58.48
850	142.69	68.24

^a^Calculated using the Scherrer equation.

The lattice parameters of the as-prepared MoO_2_ nanoparticles are listed in Table 2. The lattice parameters of the MoO_2_ phase decreased as the calcination temperature increased, which also reflects the change produced by the varying size of the MoO_2_ nanoparticles. At the calcination temperature of 900 °C, the primary phase of the sample became Mo_2_C, but small diffraction peaks of MoO_2_ could be identified. After calcination at 950 °C, the MoO_2_ nanoparticles reacted with carbon during the carbonation process to form Mo_2_C, according to Equation 1:

[1]
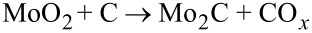


Mo_2_C is known to be active in numerous reactions associated with noble metals, such as CO_2_ hydrogenation, water gas shift, alcohol synthesis and hydrazine decomposition. Here, CH_4_/H_2_ atmosphere was not used during calcination, which was much safer and facile when compared to other methods [27].

**Table 2:** Effect of calcination temperature on BET surface area and particle size of MoO_2_.

Sample	*a* (Å)	*b* (Å)	*V* (Å^3^)

MoO_2_–CNF (550 °C)	5.6512	4.8633	132.9862
MoO_2_–CNF (650 °C)	5.6343	4.8602	132.1328
MoO_2_–CNF (750 °C)	5.6203	4.8573	131.9487
MoO_2_–CNF (850 °C)	5.6128	4.8535	131.8365
MoO_2_ (JCPDS:78-1072)	5.6500	4.8600	132.9500

Raman spectroscopy is a very useful tool for the characterization of carbon-based nanostructures. The Raman spectra of the products excited with a 532 nm laser line are shown in Figure 1b. Two characteristic peaks at around 1355 and 1580 cm^−1^ correspond to disordered carbon (D-band) and graphite carbon (G-band), respectively. Integrating of the areas of the D and G peaks yielded a significant enhancement in the corresponding *I*_G_/*I*_D_ ratio. Thus, it could be concluded that an increased calcination temperature led to the formation of significant amounts of graphitic carbon. Both the XRD and Raman spectra revealed that MoO_2_–CNF was successfully prepared through electrospinning.

**Figure 2:**
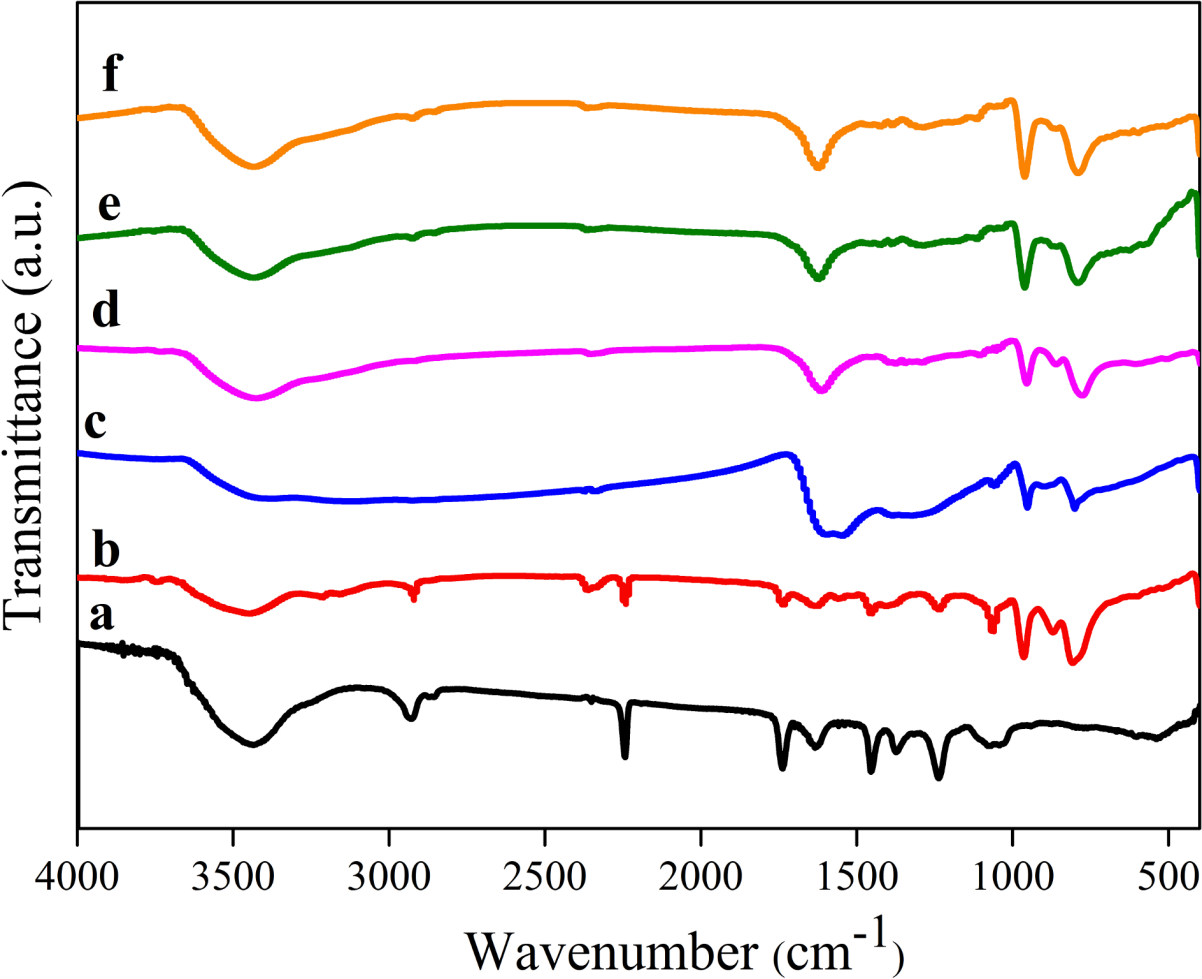
FTIR spectra of (a) PAN fibers and MoO_2_–CNFs (b) as-prepared PAN/PMA composite fibers, and (c–f) fibers calcined at 550 °C, 650 °C, 750 °C and 850 °C, respectively.

The Fourier transform infrared spectroscopy (FTIR) spectra of PAN fibers, as-prepared composite PAN/PMA fibers, and composite fibers calcined at different temperatures are illustrated in Figure 2. The FTIR spectra of PAN fibers and as-prepared PAN/PMA fibers presented characteristic absorption peaks at 2242 cm**^−^**^1^ (–C≡N) and 1736 cm**^−^**^1^ (C=O), indicating that PAN played the role of a copolymer or that the DMF solvents did not entirely volatilize (Figure 2a,b). The bands in the regions of 2934–2890, 1465–1445, 1385–1355, and 1270–1210 cm**^−^**^1^ were assigned to the aliphatic CH group vibrations of different modes in CH, CH_2_ and CH_3_. After calcination from 550 °C to 850 °C, the absorption bands of PAN vanished due to decomposition and removal of the organic groups. The peak at 925 cm**^−^**^1^ was associated with Mo=O, while the prominent bands in the range of 500–850 cm**^−^**^1^ were attributed to Mo–O–Mo, indicating the occurrence of crystallization [28]. Both the Raman spectra and XRD results suggested that MoO_2_–CNFs were formed through a subsequent annealing process. The broad band at 3400 cm**^−^**^1^ was attributed to the O–H stretching vibration due to absorbed H_2_O molecules on the nanofibers of KBr.

**Figure 3:**
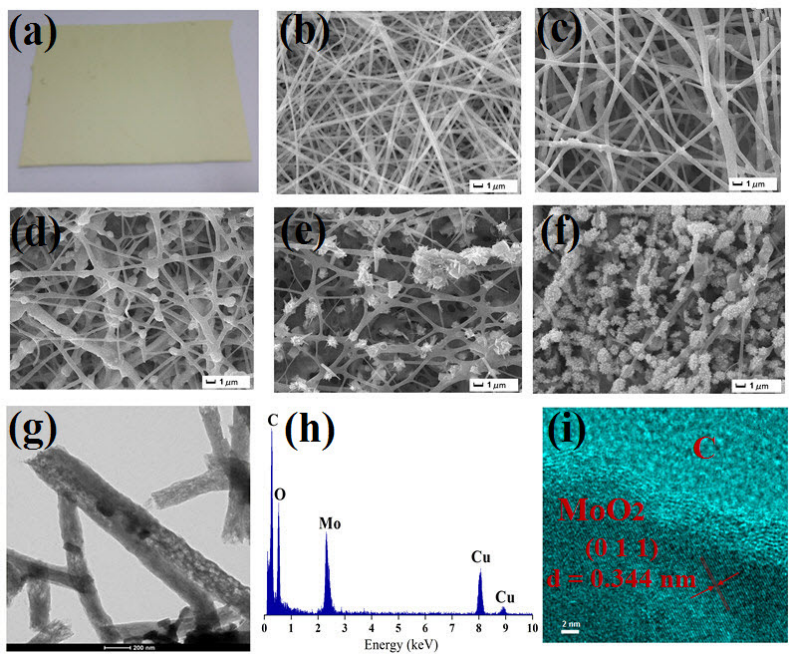
(a) A photo of nonwoven PAN/PMA fabric. SEM images of (b) as-prepared PAN/PMA composite fibers, (c-–f) fibers calcined at 550 °C, 650 °C, 750 °C and 850 °C. (g) TEM image of MoO_2_–CNF calcined at 850 °C. (h) EDX elemental line analysis and (i) HRTEM image of MoO_2_–CNFs.

A photo of the nonwoven PAN/PMA material is depicted in Figure 3a. The morphology of the as-prepared composite fibers and calcined fibers was further characterized by FE-SEM and TEM. The PAN/PMA composite fibers showed smooth surfaces due to their amorphous nature (Figure 3b). The average diameter of the as-prepared composite fibers was estimated to be 485 nm. After calcination of the fibers at 550 °C, the surface became rough and the average diameter decreased to 425 nm. The shrinkage and reduction in the fibers was caused by decomposition of PAN and subsequent crystallization. After calcination at 650 °C and 750 °C, MoO_2_–CNFs showed discrete lengths with average diameters of 506 nm and 575 nm, respectively. Also, the diameter of MoO_2_–CNF increased as the calcination temperature was increased, which can be explained by the gradual increase in grain size of MoO_2_ with sintering temperature. Interestingly, a change in fiber morphology was observed when the calcination temperature increased to 850 °C. The nanofibers consisted of connected particles or crystallites, which is consistent with previous reports [29]. Further structural characterization of the as-prepared MoO_2_–CNFs was performed by TEM. Figure 3g shows a typical TEM photograph of the nanostructures, displaying MoO_2_ nanoparticles decorated with carbon nanofibers. The elemental EDX of MoO_2_–CNFs depicted in Figure 3h indicates the presence of elemental Mo, O, C and Cu. The Cu signal comes from the Cu grid. The HRTEM image indicated that the grown structure was single crystalline with a lattice spacing of 0.344 nm, corresponding to the [11] crystal plane of monoclinic MoO_2_ (Figure 3i).

**Figure 4:**
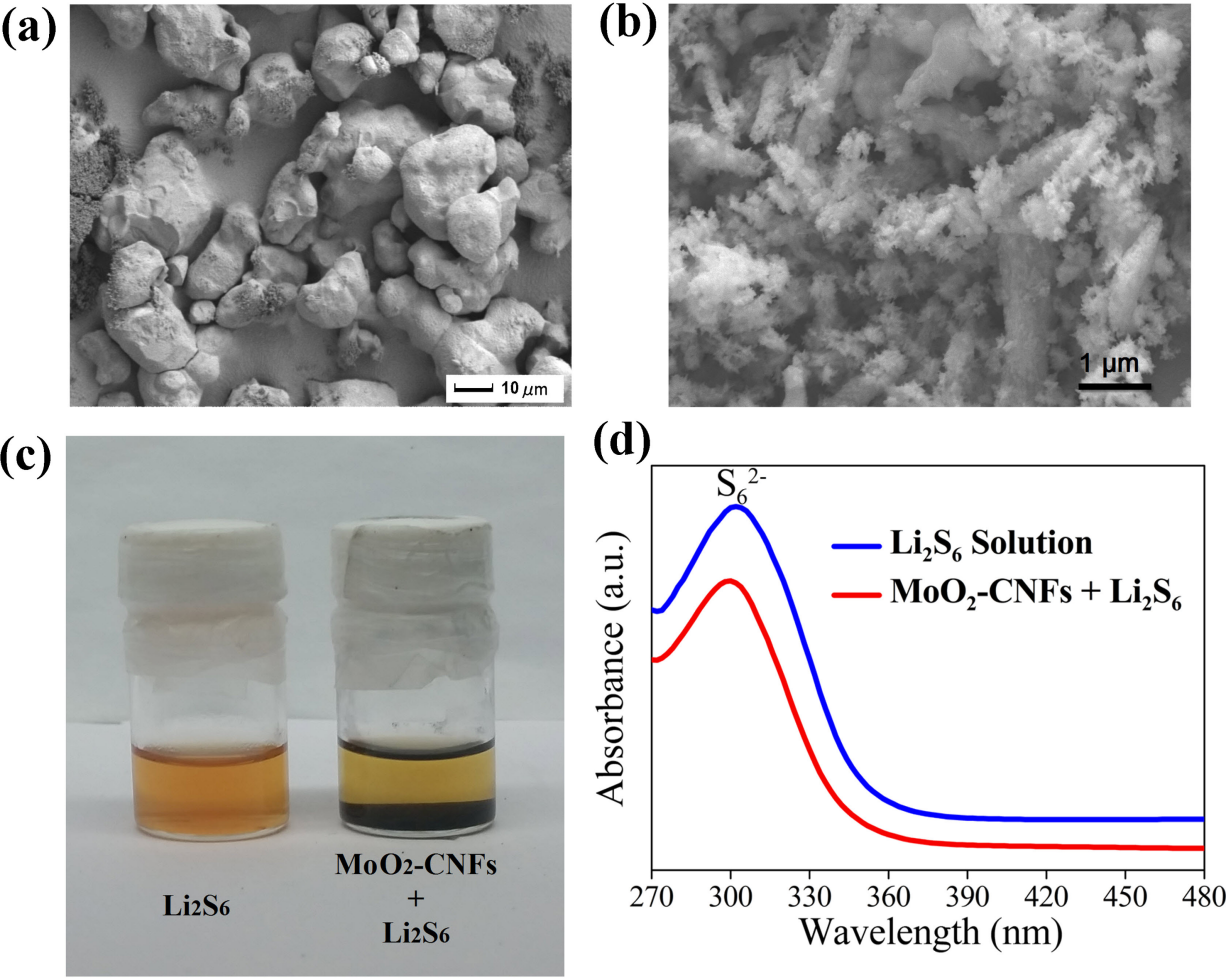
SEM image of (a) pure sulfur, (b) MoO_2_–CNF/sulfur composite. (c) Optical photo of Li_2_S_6_ adsorption on MoO_2_–CNFs and (d) UV–vis absorption spectra of the Li_2_S_6_ solution before and after the addition of MoO_2_–CNFs.

SEM images of pure sulfur and S/MoO_2_–CNF composites are displayed in Figure 4a,b, respectively. The sulfur morphology was drastically changed from smooth to rough agglomerated particles upon the addition of MoO_2_–CNFs. The MoO_2_–CNFs acted as a conductive matrix and facilitated the dispersion of sulfur with smaller particle size, which could favor ion diffusivity in the cathode due to the reduction in the Li-ion pathways. To further evaluate the interaction between MoO_2_–CNFs and polysulfides, the as-prepared MoO_2_–CNFs were added into Li_2_S_6_ solution. In the optical photo of Figure 3c, the original yellow-brown solution turned lighter, indicating a strong adsorption. Meanwhile, UV-visible absorption spectroscopy was used to analyze the change in concentration of Li_2_S_6_ before and after the addition of MoO_2_–CNFs. The polysulfide solution showed a broad absorption region between 270 and 330 nm, with characteristic peaks located at approximately 300 nm, ascribed to S_6_^2−^ species [30]. After absorption for 0.5 h, a large decrease in the absorption peak intensity of the solution with MoO_2_–CNFs at 300 nm was identified, which confirmed the improved absorption capability of the composite fibers for polysulfidies.

### Electrochemical performance of S/MoO_2_–CNF electrodes

The electrochemical performance of cells with S/MoO_2_–CNF-based electrodes were evaluated by cyclic voltammetry (CV), galvanostatic charge–discharge and electrochemical impedance spectroscopy (EIS).

**Figure 5:**
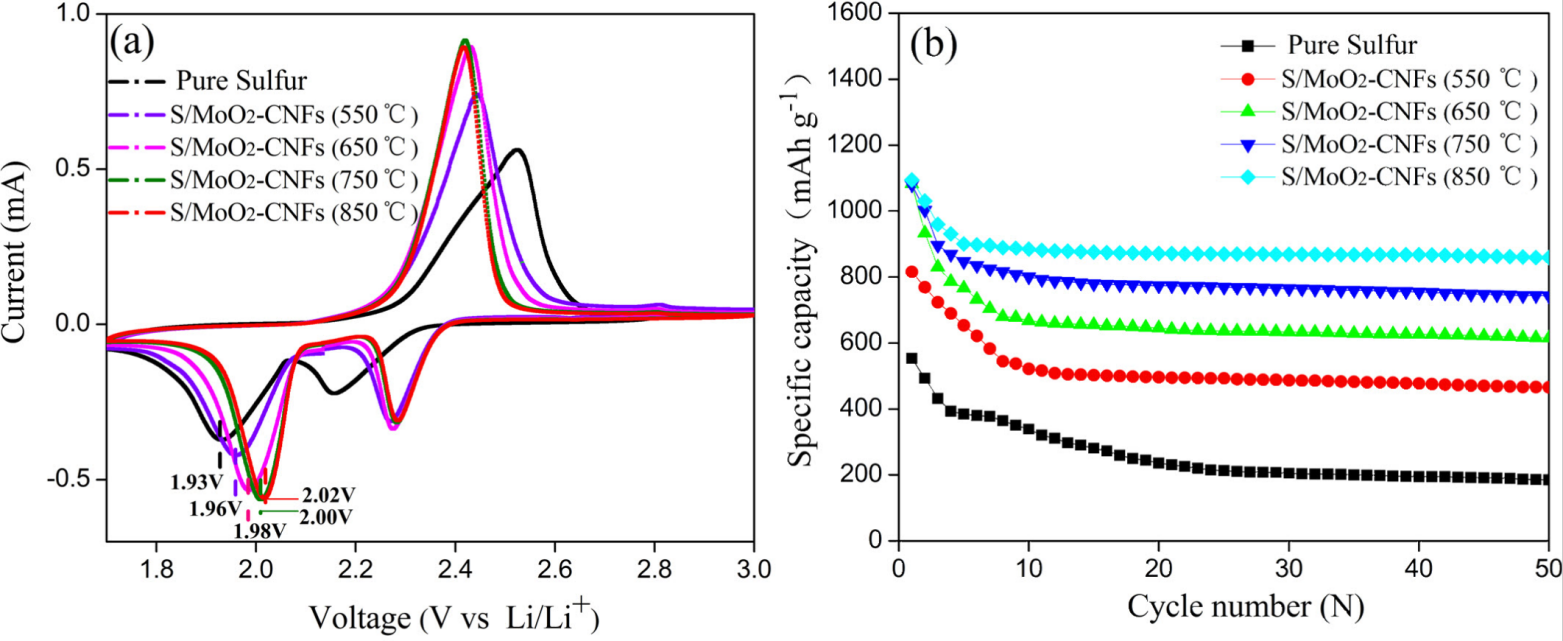
(a) The CV curves of cells assembled with S/MoO_2_–CNF cathodes and pure sulfur cathodes. (b) Cycling performance of MoO_2_–CNFs calcined at different temperatures with S/composite cathodes and pure sulfur cathode at 0.25 mA cm^−2^.

The electrochemical characteristics of the cells with S/MoO_2_–CNF cathodes and pure sulfur cathodes were examined by CV in the voltage range of 1.7–3.0 V at the scanning rate of 0.1 mV s**^−^**^1^, as shown in Figure 5a. Among these samples, all the CV curves appeared in the range of 1.93–2.05 V, 2.15–2.28 V and 2.41–2.52 V, which are typical redox reactions of Li–S batteries [31–32]. Meanwhile, the CV data confirm that the MoO_2_–CNF additive is not electrochemically active in the selected voltage region. Additionally, when comparing the CV of the pure sulfur electrode, a distinguishable positive shift in the reduction–oxidation peaks of the sulfur/MoO_2_–CNF composites can be observed, which confirms a relatively low potential polarization with MoO_2_–CNF additives. An interesting point to note is that the highest current density of the S/MoO_2_–CNF cathodes with MoO_2_–CNF calcined at 850 °C indicates enhanced reaction kinetics in the charge–discharge process. Furthermore, from Supporting Information File 1, Figure S1, the coin cell of sulfur/MoO_2_–CNF (850 °C) also showed the lowest voltage hysteresis (Δ*V*) among the cells, suggesting a highly facile electrochemical redox reaction and low resistance [33]. These findings demonstrated that MoO_2_–CNFs improve the electrochemical reaction kinetics during the charge–discharge process.

Figure 5b presents the cycling performance of the cells assembled from sulfur cathodes with and without MoO_2_–CNFs calcined at different temperatures. The cell assembled with the pure sulfur electrode revealed lower initial discharge capacity. After a few cycles, the discharge capacity reduced from 554 mAh g**^−^**^1^ to 186 mAh g**^−^**^1^. Compared to the pure sulfur cathode, the cathode performance clearly improved when MoO_2_–CNFs were present in the sulfur matrix. The initial discharge capacity of the S/MoO_2_–CNF cathodes with MoO_2_–CNFs calcined at 550, 650, 750, and 850 °C were recorded as 816, 1082, 1079, and 1095 mAh g**^−^**^1^, respectively. The improved performance with the addition of MoO_2_–CNFs could be attributed to the polysulfide adsorption and improved electrochemical reaction kinetics of MoO_2_, demonstrated by the initial specific capacity and CV curves. Meanwhile, the S/MoO_2_–CNFs (calcined at 850 °C) retained the highest capacity of 860 mAh g**^−^**^1^ after 50 cycles. The performance of the MoO_2_–CNF matrix for application in Li–S batteries is also compared with several other carbon nanofibers and metal oxides fibers (Table 3), which further demonstrates the long-life behavior of the sulfur/MoO_2_–CNF cathode.

**Table 3:** Performance comparison of MoO_2_–CNFs with other matrices for application in Li–S batteries.

Matrix	Cycle performance	Ref.

MoO_2_–CNFs	860 mAh g^−1^ 0.25 mA cm^−2^ (≈0.1 C) per 50 cycles	this work
CNFs	207 mAh g^−1^ 0.1 C per 50 cycles	[34]
CNFs	390 mAh g^−1^ 0.1 C per 100 cycles	[35]
VGCFs	335 mAh g^−1^ 0.1 C per 40 cycles	[36]
CNFs	560 mAh g^−1^ 0.1 C per 50 cycles	[37]
Mg_0.6_Ni_0.4_O fibers	435 mAg g^−1^ 0.1 C per 20 cycles	[12]

**Figure 6:**
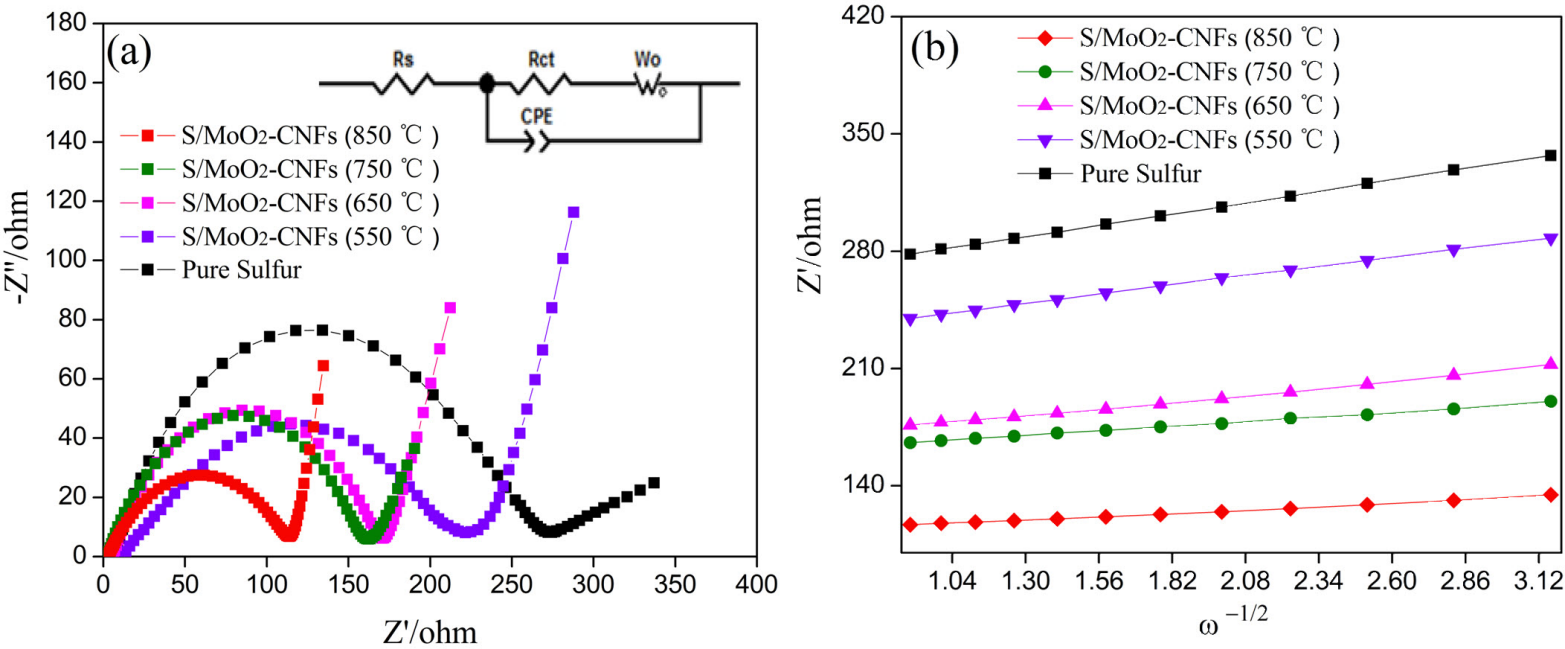
(a) Electrochemical impedance spectroscopy of MoO_2_–CNFs calcined at different temperatures with sulfur cathodes and a pure sulfur cathode. (b) The dependence of *Z*’ (*Z*_re_) on the reciprocal square root of the frequency ω^−1/2^ in the low-frequency region of five electrodes.

The EIS technique was used to investigate the effect of the MoO_2_–CNF matrix material calcined at different temperatures on the electrochemical performance of the sulfur cathode. Compared to the CV technique, the diffusion coefficients under equilibrium conditions can be expressed by electrochemical impedance spectroscopy (EIS). Additionally, the charge-transfer reaction and lithium ion diffusion in the interface of solid electrodes can be derived [38–39]. Figure 6a displays the Nyquist plots of pure sulfur and S/MoO_2_–CNFs electrodes. Each Nyquist plot consists of a semicircle in the high to medium frequency region and a sloping line in the low frequency region. The equivalent circuits compatible with the Nyquist diagrams are represented in the inset of Figure 6a, which contain the solution resistance (*R*_s_), the charge-transfer resistance of the electrode (*R*_ct_) and a constant phase element corresponding to the double-layer capacitance (CPE). A steep sloping line in the low-frequency region, corresponding to the Warburg impedance, was represented by *W*_0_. The fitting results are listed in Table 4. Obviously, the S/MoO_2_–CNF cathodes possessed lower charge transfer resistance than pure sulfur cathodes, indicating better charge transfer between the sulfur and MoO_2_–CNF materials. This suggested sufficient contact among sulfur and MoO_2_–CNFs, which lowered the resistance for the electron transfer across the interface between both. For further confirmation, the lithium ion diffusion coefficient was calculated using Equation 2 [40–41]:

[2]
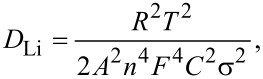


where *D*_Li_ represents the diffusion coefficient of the lithium ion, *R* is the gas constant, *T* is the absolute temperature, *A* is the surface area of electrode, *n* is the number of electrons per molecule during the reaction, *F* is the Faraday constant, *C* is the concentration of lithium ions, and σ is the Warburg factor calculated through Equation 3 [40–41],

[3]
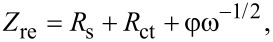


where φ is the slope of the plots and *Z*_re_ is the reciprocal root square at the lower angular frequencies (ω^−1/2^), presented in Figure 6b.

**Table 4:** Impedance parameters of the electrodes.

Electrodes	*R*_s_ (Ω)	*R*_ct_ (Ω)	*D*_Li_ (cm^2^ s**^−^**^1^)

sulfur/MoO_2_–CNF (850 °C)	1.387	113.92	8.42 × 10**^−^**^14^
sulfur/MoO_2_–CNF (750 °C)	1.584	161.15	4.38 × 10**^−^**^14^
sulfur/MoO_2_–CNF (650 °C)	2.614	169.53	2.05 × 10**^−^**^14^
suflur/MoO_2_–CNF (550 °C)	3.004	221.59	1.16 × 10**^−^**^14^
pPure sulfur	3.052	274.34	7.71 × 10**^−^**^15^

## Conclusion

MoO_2_–CNF materials were prepared using the electrospinning process of PAN/PMA mixtures, followed by calcination treatments. XRD, FTIR and Raman results suggest that MO_2_–CNFs were obtained after being calcined at 550 °C and complete removal of the inorganic compound. The SEM images showed that the as-prepared MO_2_–CNF composite fibers had a smooth surface which turned to rough after calcination, revealing the increased crystallinity of MoO_2_ associated with the rise of the calcination temperature. The obtained MoO_2_–CNFs were applied to a sulfur matrix for Li–S batteries and shown to exhibit high capacity when compared to electrodes with pure sulfur. The improved electrochemical performance could be attributed to the adsorption of polysulfide and acceleration of the electrochemical reaction kinetics during the charge–discharge process. The EIS results demonstrated that S//MoO_2_–CNF composites display a markedly higher lithium-ion diffusion coefficient, a low interfacial resistance and much better electrochemical performance than the pristine sulfur cathode. The proposed electrospinning technique might open new avenues for making promising nanofibers for practical applications.

## Experimental

### Synthesis of MoO_2_–CNFs

Phosphomolybdic acid (PMA: H_3_PO_4_·12MoO_3_), polyacrylonitrile (PAN, *M*_w_ = 150,000) and *N*,*N*-dimethylformamide (DMF) were purchased from Sinopharm Chemical Reagent Co., Ltd. All the reagents were used as-received without further purification.

In a typical procedure, a PAN solution (10 wt %) was prepared by dissolving PAN powder in DMF and stirring for 12 h. Next, PMA (3 g) was added to the above solution and vigorously stirred for 24 h at room temperature to form a sol–gel solution for further electrospinning. The solution was then loaded into 10 mL plastic syringes equipped with a 9-gauge stainless steel needle. A high voltage power supply was used to provide a voltage of 15 kV to the needle tips and the rotating drum collector covered by aluminum foil served as the counter electrode. The distance between the needle tips and drum collector was set to 18 cm and the flow rate of the solution to 0.5 mL h**^−^**^1^. The as-prepared electrospun nanofibers were preoxidized at 260 °C for 2 h in air and calcined at different temperatures for 4 h under argon atmosphere. Scheme S1 in Supporting Information File 1 illustrates the procedure used for preparing MoO_2_–CNFs.

### Preparation of S/MoO_2_–CNF electrodes

Sulfur/MoO_2_–CNF (S/MoO_2_–CNF) composites were prepared by mixing sulfur and MoO_2_–CNFs in a mortar at the weight ratio of 1:1. The resulting S/MoO_2_–CNF composites were gradually dried in air for 6 h then heated to 155 °C for 6 h in a sealed 25 mL teflon-lined stainless-steel autoclave. After cooling down to room temperature, S/MoO_2_–CNF composites were obtained. Next, the as-prepared S/MoO_2_–CNF composites were mixed with acetylene black and polyvinylidene fluoride (PVDF) in *N*-methyl-2-pyrrolidone (NMP) at a weight ratio of 7:2:1. The slurry was spread onto aluminum foil (thickness: 20 μm) and dried in vacuum at 60 °C for 12 h. Electrodes were made from punching circular discs with a diameter of 12 mm and sulfur loadings of 1.5 mg cm^−2^ were applied. The thickness of the electrodes was 35 μm. For comparison, a pure sulfur cathode was prepared using the same procedure by mixing sulfur, acetylene black and PVDF at the weight ratio of 7:2:1. The S/MoO_2_–CNF electrode is schematically displayed in Scheme S2 of Supporting Information File 1.

### Materials characterization

The crystalline phases of the samples were determined by X-ray diffraction (XRD, Rigaku D/Mmax 2500PC) using Cu Kα radiation (λ = 1.5406 Å). The average grain size (*D*) of the MoO_2_ nanoparticles was calculated using the Scherrer equation (*D* = 0.89λ/(βcosθ)), where λ represents the wavelength of the X-ray diffraction, β is the full width at half maximum of the relevant diffraction peak, and θ is the diffraction angle. The Raman spectra were recorded on an American Themo-Fisher spectrometer using an Ar^+^ laser at 532 nm. The Brunauer–Emmett–Teller (BET) surface area was determined by nitrogen adsorption–desorption using a NOVA 2000e analyzer. The presence of functional groups was examined by Fourier transform infrared spectrometry (FTIR, Avatar-370 spectrometer) using the standard method of KBr in the scanning range of 400–4000 cm**^−^**^1^. The size and morphology of the fibers was determined by scanning electron microscopy (SEM, JSM-7001F). Details concerning the morphology and structure were examined by high-resolution transmission electron microscopy (HRTEM, Tecnai G2 F30), operated at an accelerating voltage of 200 kV. Selected specimens were examined with energy dispersive X-ray (EDX) spectroscopy and elemental mapping attached to the HRTEM operating at 200 kV.

The adsorption ability was determined by preparing a Li_2_S_6_ solution through the addition of Li_2_S to sulfur at the molar ratio of 1:5 in tetrahydrofuran (THF) under stirring. The obtained solution containing about 1.8 mg mL**^−^**^1^ Li_2_S_6_ was used for the sulfide adsorption test. MoO_2_–CNFs were added to 10.0 mL of Li_2_S_6_/TFH solution and the mixture was adequately stirred for 0.5 h. The ability of the MoO_2_–CNF composite to adsorb Li_2_S_6_ was evaluated by UV–vis spectroscopy (UV-1800PC, Shanghai Mapada Instrument Co. Ltd).

### Electrochemical measurements

The electrochemical performance of the samples was measured in CR 2032-type coin cells. The electrolyte contained 1 M lithium bis(trifluoromethanesulfone)imide (LITFSI) and 0.1 M LiNO_3_ dissolved in 1,3-dioxolane (DOL) and 1.2-dimethoxyethane (DME) at a volume ratio of 1:1. The electrolyte solution volume used in the cells was 75 μL. The coin cells were galvanostatically charged–discharged at 0.25 mA/cm^2^ (1 C = 1675 mA g^−1^) and a voltage ranging from 1.7 and to 3.0 V (vs Li/Li^+^) using a CT2001A cell test instrument (LAND model, Wuhan RAMBO testing equipment, Co. Ltd.). The CV and EIS measurements were conducted on a VMP2 electrochemical workstation (DHS Instruments Co. Ltd.). The CV curves were recorded at a scan rate of 0.1 mV s^−1^ in the voltage range of 1.7–3.0 V. The EIS spectra were measured in the frequency range of 0.1–100 kHz with a disturbance amplitude of 10 mV.

## Supporting Information

File 1 Additional experimental data and experimental schemes.
