# Supplementary material for: Synthesis and characterization of electrospun molybdenum dioxide–carbon nanofibers as sulfur matrix additives for rechargeable lithium–sulfur battery applications
Source: Beilstein J Nanotechnol. 2018 Jan 24;9:262–70. doi: 10.3762/bjnano.9.28 (PMC5789398; doi:10.3762/bjnano.9.28)
Supplement: File 1 — Additional experimental data and experimental schemes. [file Beilstein_J_Nanotechnol-09-262-s001.pdf]

Supporting Information

for

Synthesis and characterization of electrospun molybdenum dioxide–carbon nanofibers as sulfur matrix additives for rechargeable lithium–sulfur battery applications

Ruiyuan Zhuang[1‡], Shanshan Yao*[1‡], Maoxiang Jing[1], Xiangqian Shen[1,2], Jun Xiang[3], Tianbao Li[2], Kesong Xiao[2] and Shibiao Qin[2]

Address: [1]Institute for Advanced Materials, College of Materials Science and Engineering, Jiangsu University, Zhenjiang, 212013, P. R. China, [2]Hunan Engineering Laboratory of Power Battery Cathode Materials, Changsha Research Institute of Mining and Metallurgy, Changsha, 412212, P. R. China and [3]School of Mathematics and Physics, Jiangsu University of Science and Technology, Zhenjiang, 212013, P. R. China

Email: Shanshan Yao*-yaosshan@ujs.edu.cn

* Corresponding author

[‡]These authors contributed equally to this work.

Additional experimental data and experimental schemes

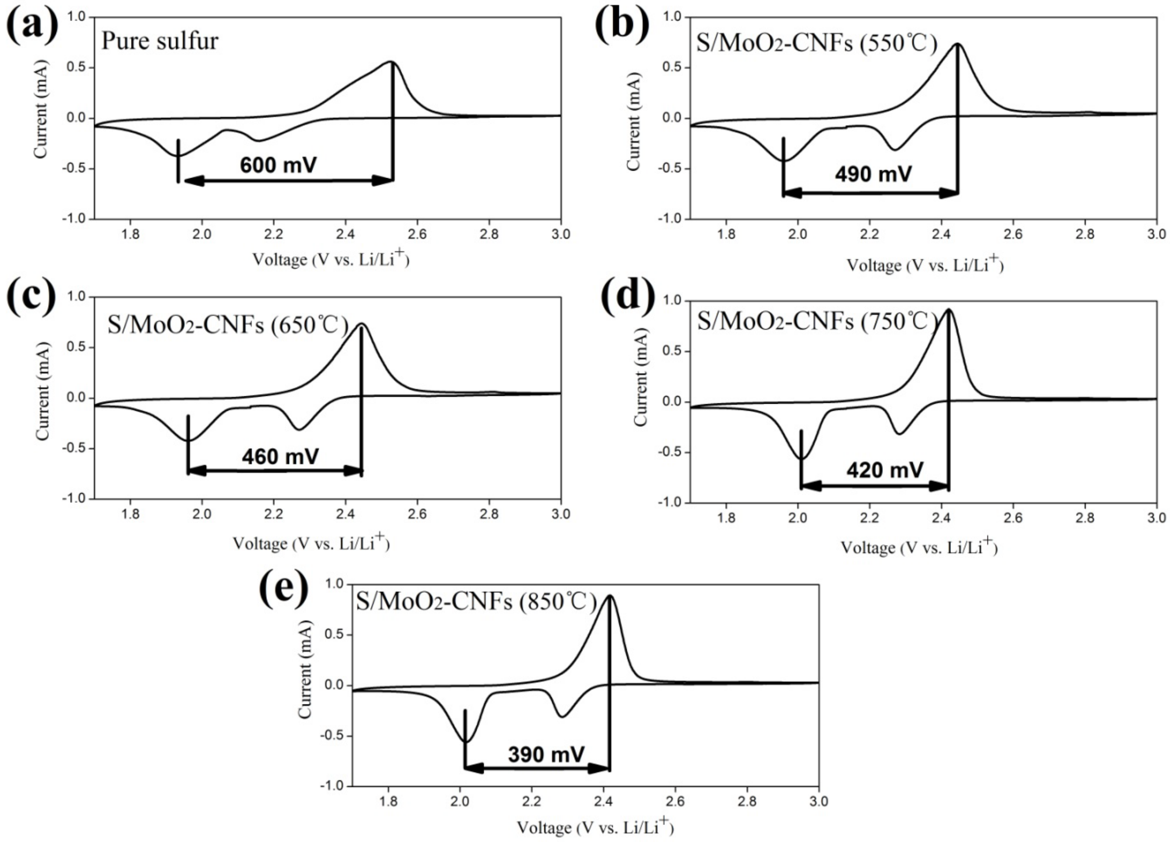

Figure S1: CV curves of Li-S cells with (a) pure sulfur electrode, (b-e) sulfur/MoO_2–CNF composite electrodes.

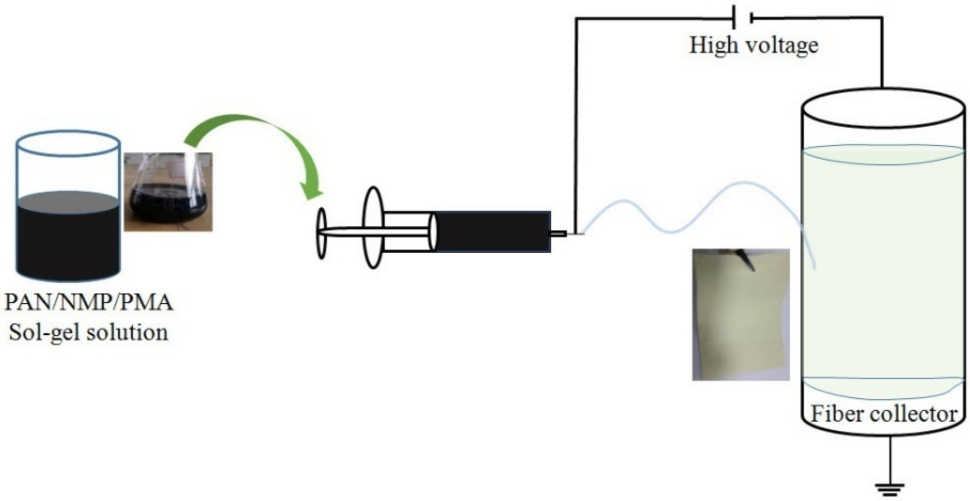

Scheme S1: The schematic illustration of the procedure used to prepare MoO_2–CNFs.

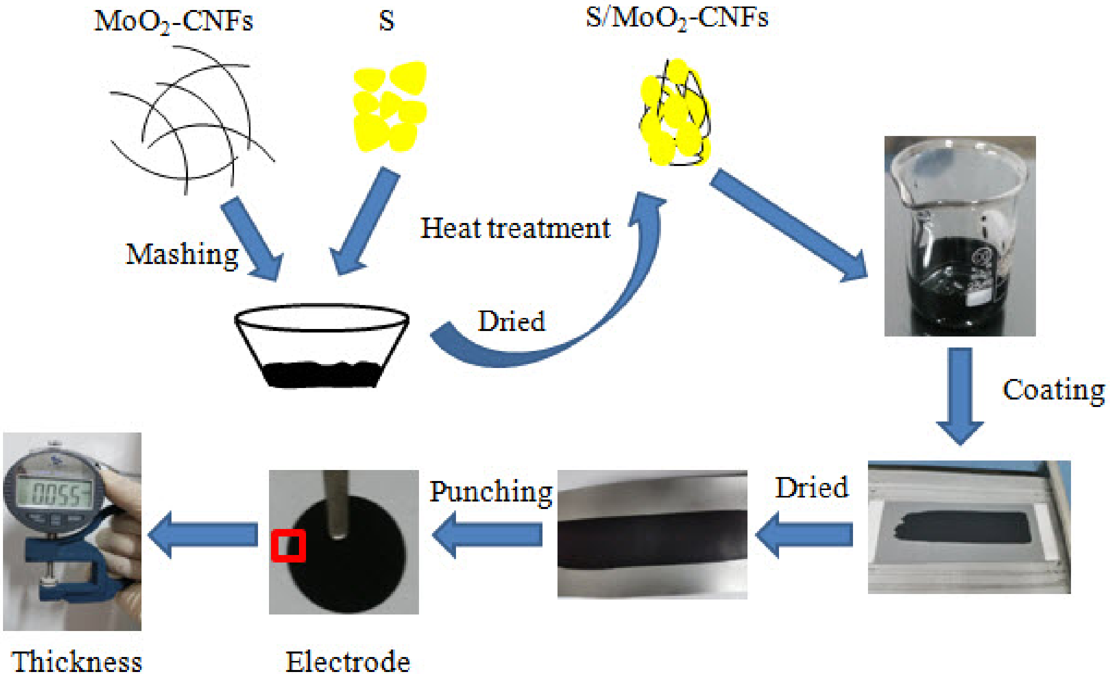

Scheme S2: A schematic representation of the S/MoO_2–CNF electrodes.
